# Environmental enrichment modulates parvalbumin interneuron deficits and plasticity-related signaling after early postnatal NMDA receptor hypofunction in male rat visual cortex

**DOI:** 10.1007/s00429-026-03168-8

**Published:** 2026-08-04

**Authors:** Ane Murueta-Goyena, Naiara Ortuzar, Susana Bulnes, José Vicente Lafuente, Harkaitz Bengoetxea

**Affiliations:** 1 LaNCE-Neuropharm, Department of Neurosciences, Faculty of Medicine and Nursery, University of the Basque Country (EHU), 48940 Leioa, Bizkaia Spain; 2 Neurodegenerative diseases group, Biobizkaia Health Research Institute, 48903 Barakaldo, Bizkaia Spain

**Keywords:** Schizophrenia, Parvalbumin, N-methyl-D-aspartate antagonists, Visual Cortex, Environmental enrichment

## Abstract

Sensory processing deficits in schizophrenia have been linked to dysfunction of cortical inhibitory interneurons, particularly parvalbumin-expressing (PV+) populations. NMDA receptor hypofunction during development is known to disrupt interneuron maturation, but its long-term impact on visual cortex circuitry and the potential for experience-dependent modulation in adulthood remain unclear. Here, we examined the effects of early postnatal N-methyl-D-aspartate (NMDA) receptor blockade with MK-801 on the number of PV+ and somatostatin-expressing (SST+) interneurons in the primary visual cortex (V1) and assessed whether environmental enrichment (EE) in adulthood modulates these alterations by analyzing molecular changes using Western blot. Male Long–Evans rats received MK-801 (0.5 mg/kg) or saline from postnatal day 10–20, followed by EE exposure from P55–73. Stereological analyses revealed a marked reduction of PV-immunoreactive cells in layers II/III and IV, while SST+ populations were largely preserved. EE increased the number of PV-immunoreactive cells across groups, and modestly enhanced SST+ cells in layer IV, although no treatment × housing interaction was detected, indicating a general enrichment-related effect. At the molecular level, MK-801 reduced expression of the NMDA receptor subunit NR1 and increased Akt phosphorylation, whereas EE enhanced PSD95 expression, ERK phosphorylation, and GABA_A_ β2/3 subunit levels, without increasing NR1 levels. These findings indicate that early NMDA receptor hypofunction induces long-lasting, subtype-specific alterations in inhibitory circuitry in V1. EE in adulthood engages molecular pathways associated with synaptic plasticity and modulates interneuron immunoreactivity, suggesting that inhibitory circuits retain some capacity for experience-dependent remodeling despite persistent receptor-level deficits.

**Supplementary Information:**

The online version contains supplementary material available at https://doi.org/10.1007/s00429-026-03168-8.

## Introduction

Early sensory processing abnormalities are increasingly recognized as a core component of schizophrenia pathophysiology. In particular, converging behavioral, electrophysiological, and neuroimaging evidence indicates that dysfunction of the visual system emerges at or before illness onset and persists throughout the course of the disorder (Butler et al. 2001, 2005, 2007; Sklar et al. 2020). Importantly, alterations in the primary visual cortex (V1) and related visual regions have been associated with both positive and negative symptoms, as well as with deficits in perceptual organization and higher-order cognition (Reavis et al. 2016; Türközer et al. 2019; Sklar et al. 2020). Structural studies additionally demonstrate reduced cortical thickness in functionally defined visual areas, a feature associated with impaired perceptual performance and task-related activation in patients (Reavis et al. 2016).

At the cellular level, growing evidence implicates cortical inhibitory interneurons underlie these early visual processing deficits (Yang et al. 2011; Gagné et al. 2015). Parvalbumin-expressing (PV+) interneurons are critical regulators of cortical excitation–inhibition balance and are essential for the generation of gamma-band oscillations that support sensory processing and perceptual integration in V1. Post-mortem and neurophysiological studies indicate that schizophrenia is associated with reduced density and altered function of PV+ interneuron terminals in both primary and association visual cortices (Fish et al. 2021). Such alterations likely contribute to a reduction in inhibitory surround mechanisms, as demonstrated by population receptive field studies showing diminished inhibitory modulation in the visual cortex of individuals with schizophrenia (Anderson et al. 2017). Consistent with these cellular findings, impaired gamma-band oscillations in visual cortex—whose generation is critically dependent on PV+ interneuron function—have been reported in individuals at clinical high risk for psychosis and in patients experiencing first-episode psychosis (Grent-‘t-Jong et al. 2020), and these alterations correlate with cognitive and functional deficits (McCutcheon et al. 2020; Sohal 2022; Marín 2024).

While PV+ interneurons have received considerable attention in schizophrenia research, cortical inhibitory function depends on the coordinated activity of multiple interneuron subtypes. Among these, somatostatin-expressing (SST+) interneurons are particularly relevant because of their role in dendritic inhibition and the modulation of cortical oscillations underlying sensory processing. Converging evidence indicates that reductions in somatostatin expression in schizophrenia weaken dendritic inhibitory control over pyramidal neurons, thereby disrupting the integration of incoming visual information (Liguz-Lecznar et al. 2022; Dienel et al. 2025). Beyond this structural role, SST+ interneurons are critical for shaping electrophysiological markers of perception, including mismatch negativity, as well as for maintaining theta/alpha-band oscillatory dynamics and visual discrimination fidelity (Cottam et al. 2013; Hamm and Yuste 2016; Song et al. 2020). Although comparatively less studied than PV populations, these findings suggest that SST+ interneuron dysfunction may contribute to impaired visual processing and related perceptual disturbances in schizophrenia through combined effects on dendritic computation, oscillatory coordination, and excitation–inhibition balance (Van Der Hoorn et al. 2014; Veit et al. 2017).

Animal models have provided important insight into the mechanisms linking neurodevelopmental disruption, interneuron dysfunction, and schizophrenia-like phenotypes. Among these models, pharmacological blockade of NMDA receptors during early postnatal development with non-competitive antagonist MK-801 has been widely used to mimic aspects of the neurodevelopmental disturbances associated with schizophrenia (Lim et al. 2012; Li et al. 2015; Plataki et al. 2021). Early-life NMDA receptor hypofunction can disrupt cortical maturation, alter excitatory–inhibitory balance, and lead to persistent changes in interneuron populations and cortical circuitry (Braun et al. 2007; Lim et al. 2012; Li et al. 2015; Huang et al. 2021; Plataki et al. 2021). However, while several studies have examined the consequences of such manipulations in prefrontal and hippocampal circuits, studies examining long-term interneuron changes in sensory cortices, and specifically in V1, after early postnatal MK-801 administration are notably absent. Addressing this gap may improve our understanding of whether neurodevelopmental disruptions extend to primary sensory circuits, which are increasingly implicated in schizophrenia pathophysiology and may contribute to its cognitive manifestations.

Environmental factors can also modulate cortical circuitry and may partially counteract neurodevelopmental disruptions associated with schizophrenia. Environmental enrichment (EE) has been shown to induce structural and functional plasticity in sensory cortices, including the visual cortex. For example, exposure to EE during development increases the size of the primary visual cortex, expands visual field representation, and modifies cortical organization in mice, suggesting enhanced sensory-driven plasticity (Bengoetxea et al. 2008; Argandoña et al. 2009; Bibollet-Bahena et al. 2023). These structural adaptations are accompanied by improvements in sensory processing and cortical responsiveness. EE has also been shown to restore excitatory–inhibitory balance and synaptic plasticity in cortical networks by modulating interneuron activity. In particular, EE selectively enhances PV+ interneuron function and ameliorates synaptic and behavioral deficits in animal models of schizophrenia-like phenotypes (Huang et al. 2021). In other brain regions, EE-induced interneuron plasticity has been linked to remodeling of glutamatergic synapses and NMDA/AMPA receptor subunit composition alongside synaptic scaffolding proteins (Murueta-Goyena et al. 2019; Pintori et al. 2024), and recovery of hippocampal–prefrontal long-term potentiation (Murueta-Goyena et al. 2019). These effects are accompanied by normalization of GABAergic markers, including PV and GAD67 (Murueta-Goyena et al. 2018), re-establishment of excitatory–inhibitory balance through PV interneuron activity (Huang et al. 2021), and modulation of astrocytic and inflammatory pathways that support synaptic function (Rahati et al. 2016; Gonçalves et al. 2026). However, whether similar plasticity mechanisms operate in the V1 following early-life NMDA receptor blockade remains unknown.

Importantly, schizophrenia is widely considered a neurodevelopmental disorder in which early-life disturbances precede the clinical onset of symptoms by many years, with psychotic and cognitive manifestations typically emerging during late adolescence or early adulthood (Van Os and Kapur 2009). Accordingly, EE was initiated in early adulthood in rats to approximate the developmental stage at which schizophrenia symptoms typically emerge in humans. Previous studies from our group have shown that this paradigm induces behavioral, synaptic, and interneuron-related plasticity in the same developmental MK-801 model (Murueta-Goyena et al. 2018, 2019, 2020).

To explore these mechanisms, we focused on molecular markers representing complementary aspects of excitatory–inhibitory balance and experience-dependent plasticity. NR1 was selected as an obligatory NMDA receptor subunit, providing an index of persistent NMDA receptor involvement after developmental MK-801 exposure. PSD95 was analyzed as a postsynaptic scaffolding protein enriched at glutamatergic synapses, reflecting excitatory postsynaptic organization and the structural stabilization of glutamatergic synapses. GABA_A_ receptor β2/3 subunits are key components of postsynaptic inhibitory receptors mediating fast GABAergic transmission and may therefore provide information about inhibitory receptor availability. Finally, extracellular signal-regulated kinase 1/2 (ERK1/2) and protein kinase B (Akt) were assessed as intracellular signaling pathways involved in neuronal survival, synaptic remodeling, receptor trafficking, and activity-dependent plasticity, all of which may be influenced by both developmental NMDA receptor disruption and EE. Together, these markers allowed us to examine whether adult EE engages synaptic and intracellular signaling mechanisms in V1 despite persistent developmental NMDA receptor disruption.

Therefore, the present study sought to determine the long-term impact of early postnatal MK-801 chronic exposure (postnatal days 10 to 20) on PV- and SST-immunoreactive interneuron populations in the male rat V1 using stereological approaches. In parallel, we examined whether short-term EE initiated in adulthood could mitigate these alterations, and assessed associated molecular changes in NMDA receptor expression, excitatory postsynaptic organization, inhibitory receptor availability, and intracellular plasticity-related signaling.

## Materials and methods

### Animals

A total of 48 male Long–Evans rats were used in this study. Animals were housed under controlled environmental conditions (22 ± 1 °C; 12 h light/dark cycle) with food and water available *ad libitum*. All experimental procedures were conducted in accordance with the European Communities Council Directive (2010/63/EU) for the care and use of laboratory animals and were approved by the Ethical Committee and Animal Welfare of the University of the Basque Country (EHU) [M20_2020_282].

The animals used in the present study correspond to the same experimental cohort previously described (Murueta-Goyena et al. 2018, 2020). Brain tissue from these animals had been used previously for hippocampal and prefrontal analyses. In those studies, dorsal hippocampal sections spanned approximately Bregma − 2.40 mm to − 5.76 mm. The present V1 analysis sampled sections from Bregma − 4.68 mm to − 7.44 mm using the same section sampling factor of 1/10. Thus, a small number of previously processed sections containing rostral V1 were re-examined for the present stereological quantification together with additional sections from the same animals. No additional animals were generated or sacrificed for this study.

### Experimental procedures

To induce a neurodevelopmental disruption model associated with schizophrenia-like alterations, animals received systemic administration of the non-competitive NMDA receptor antagonist MK-801 (dizocilpine hydrogen maleate) purchased from Sigma-Aldrich (Cat# M107). MK-801 was dissolved in sterile saline, and a dose of 0.5 mg/kg was administered intraperitoneally once daily during the early postnatal period, from postnatal day (P) 10 to P20. Control animals received equivalent injections of saline during the same developmental window.

**Fig. 1:**
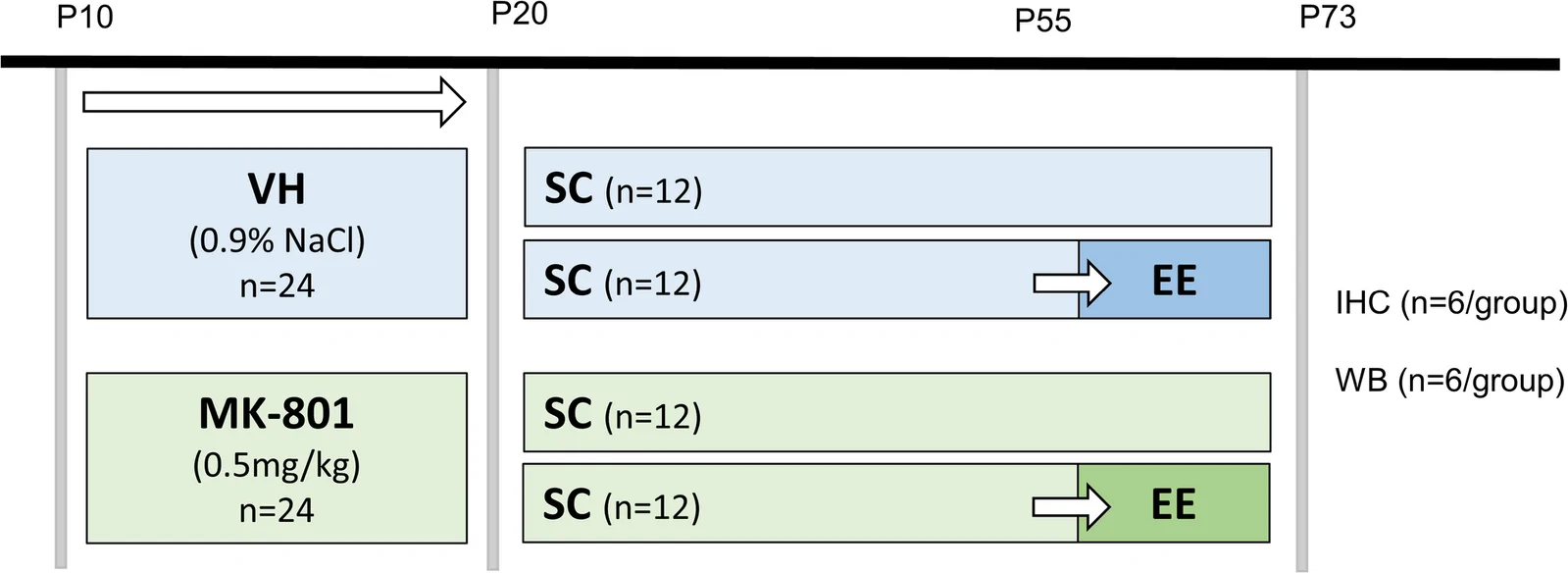
Experimental design. Schematic diagram depicting animal groups, MK-801 or saline injection schedule, timing of enriched environment (EE) housing, and endpoint for tissue collection (*n* = 12 per group). *EE* enriched environment, *MK-801* animals receiving intraperitoneal MK-801 injections (0.5 mg/kg) once daily from P10 to P20, *SC* standard conditions, *VH* vehicle-injected controls receiving saline once daily from P10 to P20

Newborn Long Evans rats were allocated to four experimental groups (*n* = 12 per group) according to treatment protocol and housing conditions (Fig. 1). Animals receiving saline injections between P10-P20 were designated as the vehicle group (VH), whereas those administered MK-801 during the same developmental period were classified as the MK-801 group. All animals were maintained under standard laboratory housing conditions from P0 to P55 in cages measuring 500 mm × 280 mm × 140 mm. From P55 to P73, EE was introduced for the designated groups (one VH group and one MK-801 group). The EE involved housing animals in larger cages (720 mm × 550 mm × 300 mm) that provided continuous access to running wheels to enable voluntary physical activity. Sensory stimulation was enhanced through the inclusion of objects with varying shapes and colors (e.g., shelters, tunnels, and toys), which were replaced every two days to maintain novelty. Social interaction was also encouraged by group housing, with six animals placed in each cage. Therefore, the following experimental groups were used: VH, MK-801, MK-801 + EE, and VH + EE.

### Immunohistochemistry

Immunohistochemical analyses were performed in six animals from each experimental condition. On P73, rats were deeply anesthetized with sodium pentobarbital prior to transcardial perfusion with 0.9% sodium chloride solution followed by 4% paraformaldehyde in 0.1 M phosphate-buffered saline (PBS, pH 7.4). Brains were subsequently removed and post-fixed overnight in the same fixative at 4 °C and then stored in 30% sucrose solution at 4 °C for cryoprotection.

Serial coronal brain sections (50 μm thickness) were obtained using a cryostat (Leica, Wetzlar, Germany). Antigen retrieval was performed for SST immunohistochemistry in sodium citrate buffer (pH 6.0) heated to 100 °C for 10 min, washed two times for 5 min in 0.1 M PBS. Then, all free-floating sections were washed three times for 5 min in 0.1 M PBS and then incubated for 20 min in 3% hydrogen peroxide to quench endogenous peroxidase activity. After three washes in PBS, sections were blocked with 5% normal horse serum (NHS) in 0.1 M PBS containing 0.5% Triton X-100 (PBS-TX) for 1 h.

Sections were then incubated in a blocking solution overnight at 4 °C with the corresponding primary antibody (mouse anti-parvalbumin, 1:5000, Swant Cat# PV235, RRID: AB_3698492; rabbit anti-somatostatin, 1:5000, Peninsula Laboratories Cat# T-4103.0050, RRID: AB_518614). The following day, after additional washing steps, sections were incubated with the secondary antibody (horse anti-mouse IgG, Vector Laboratories Cat# PK-6102, RRID: AB_2336821 or horse anti-rabbit IgG, Vector Laboratories Cat# PK-6200, RRID: AB_2336826) diluted 1:200 in PBS-TX for 1 h at room temperature. After three washes in 0.1 M PBS (5 min each), sections were incubated with the avidin–biotin complex (Vectastain Elite ABC Kit, Vector Laboratories) and immunoreactivity was visualized using 3,3′-diaminobenzidine (DAB; Sigma-Aldrich, Cat# D5637) as the chromogen. Finally, sections were mounted onto slides, air-dried, cleared in xylene for 2 h, and coverslipped with DPX mounting medium (Sigma-Aldrich, Cat# 06522).

### Stereological quantification

Quantification of PV and SST-immunoreactive cells was performed using unbiased stereological methods based on the optical fractionator principle. Analyses were conducted using a computer-assisted stereology system (Mercator Image Analysis system, Explora-Nova, La Rochelle, France) connected to a Olympus BX41 light microscope equipped with a motorized stage and high-resolution digital camera, as previously described (Murueta-Goyena et al. 2018, 2020). The boundaries of V1 were delineated at low magnification (4×) based on cytoarchitectonic criteria, spanning from Bregma − 4.68 mm to − 7.44 mm, with reference to the Paxinos and Watson stereotaxic atlas. Within this region, stereological sampling was restricted to layer II/III and layer IV, which were identified based on laminar architecture.

Systematic random sampling was applied throughout the region of interest. The section sampling factor (ssf) used in this study was 1/10. The counting frame size was 80 × 80 μm, and the spacing between optical dissectors was 120 × 120 μm, resulting in a grid size of 200 × 200 μm. A guard zone of 5% was applied, and cells were counted at 40× magnification according to established stereological principles. Sampling parameters were adjusted to achieve a coefficient of error (CE; Gundersen, m = 1) below 0.10 whenever possible. The stereological estimate for PV+ interneurons from one animal in the MK-801 + EE group showed a CE of 0.36, exceeding commonly accepted precision limits, and was therefore excluded from statistical analysis. Sensitivity analysis indicated that inclusion of this estimate did not alter the direction or significance of the main effects. Volume estimations were obtained using Cavalieri’s method.

### Western blot analysis

Western blot analyses were performed in six animals from each experimental condition. The primary visual cortices of adult Long–Evans rats (P73) were dissected and homogenized in RIPA lysis buffer (50 mM Tris-HCl, 150 mM NaCl, 0.5% sodium deoxycholate, 0.1% sodium dodecyl sulfate, 1% Triton X-100) containing a protease inhibitor cocktail (Sigma-Aldrich, Cat# P3840). Total protein concentration in each sample was determined using the Bradford protein assay (Bio-Rad, Cat# 5000006). Relative expression levels of the following proteins were quantified in visual cortex: NMDAR subunit NR1 (mouse anti-NR1, 1:1000, Millipore Cat# 05–432, RRID: AB_390129), postsynaptic density-95 (rabbit anti-PSD-95, 1:1000, Frontier Institute Cat# PSD95-Rb, RRID: AB_2571611), phosphorylated Akt (rabbit anti-pAkt (Ser 473), 1:1000, Cell Signaling Technology Cat# 9271, RRID: AB_329825); total Akt (rabbit anti-Akt, 1:1000, Cell Signaling Technology Cat# 9272, RRID: AB_329827); phosphorylated extracellular signal-regulated kinase-1/2, 1:1000, (rabbit anti-phospho-MEK1/2, Cell Signaling Technology Cat# 2338, RRID: AB_490903), total ERK1/2 (rabbit anti-p44/42 MAPK (Erk 1/2), 1:1000, Cell Signaling Technology Cat# 9102, RRID: AB_330744); GABA_A_ receptor ß2/3 subunit (mouse anti-GABA_A_ receptor ß2/3, 1:1000, Millipore Cat# 05–474, RRID: AB_309747). β-actin was used as a loading control (rabbit anti-actin,1:2000; Sigma-Aldrich Cat# A2066, RRID: AB_476693).

Equal amounts of protein were separated by SDS–polyacrylamide gel electrophoresis (SDS-PAGE) and transferred onto PVDF membranes using a semi-dry transfer system (Trans-Blot^®^ Turbo™, Bio-Rad). Membranes were blocked for 2 h at room temperature in TTBS buffer (100 mM Tris-HCl, 0.9% NaCl, 1% Tween-20, pH 7.4) containing 5% non-fat dry milk to prevent nonspecific binding. Membranes were then incubated overnight at 4 °C with the above-mentioned primary antibodies. The following day, membranes were incubated with HRP-conjugated secondary antibodies at a dilution of 1:20,000 (anti-rabbit IgG, Sigma-Aldrich Cat# 12–348, RRID: AB_390191; or anti-mouse IgG, Sigma-Aldrich Cat# 12–349, RRID: AB_390192) for 1 h at room temperature. Immunoreactive bands were visualized using a chemiluminescent detection system (SuperSignal^®^ West Dura Extended Duration Substrate, Fisher Scientific, Cat# 34076). Images were acquired using the ChemiDoc™ XRS+ Imaging System (Bio-Rad), and band densities were quantified using Image Studio Digits 3.1 software.

### Statistical analysis

Data are presented as mean ± standard deviation. Statistical analyses were performed using RStudio software (version 2024.09.0). A two-way analysis of variance (ANOVA) was conducted to assess differences between experimental groups, with treatment (saline vs. MK-801) and housing condition (standard vs. enriched environment) as fixed factors. The interaction term (treatment × housing) was included to evaluate potential combined effects. Model assumptions were verified by assessing the normality and homogeneity of residuals. When these assumptions were not met, dependent variables were log-transformed prior to analysis. Coefficient of variations of stereological estimates were calculated and compared between groups with Brown–Forsythe test. Statistical significance was set at *p* < 0.05.

## Results

### Vulnerability of PV+ interneurons and modulation by environmental enrichment in the primary visual cortex

**Fig. 2:**
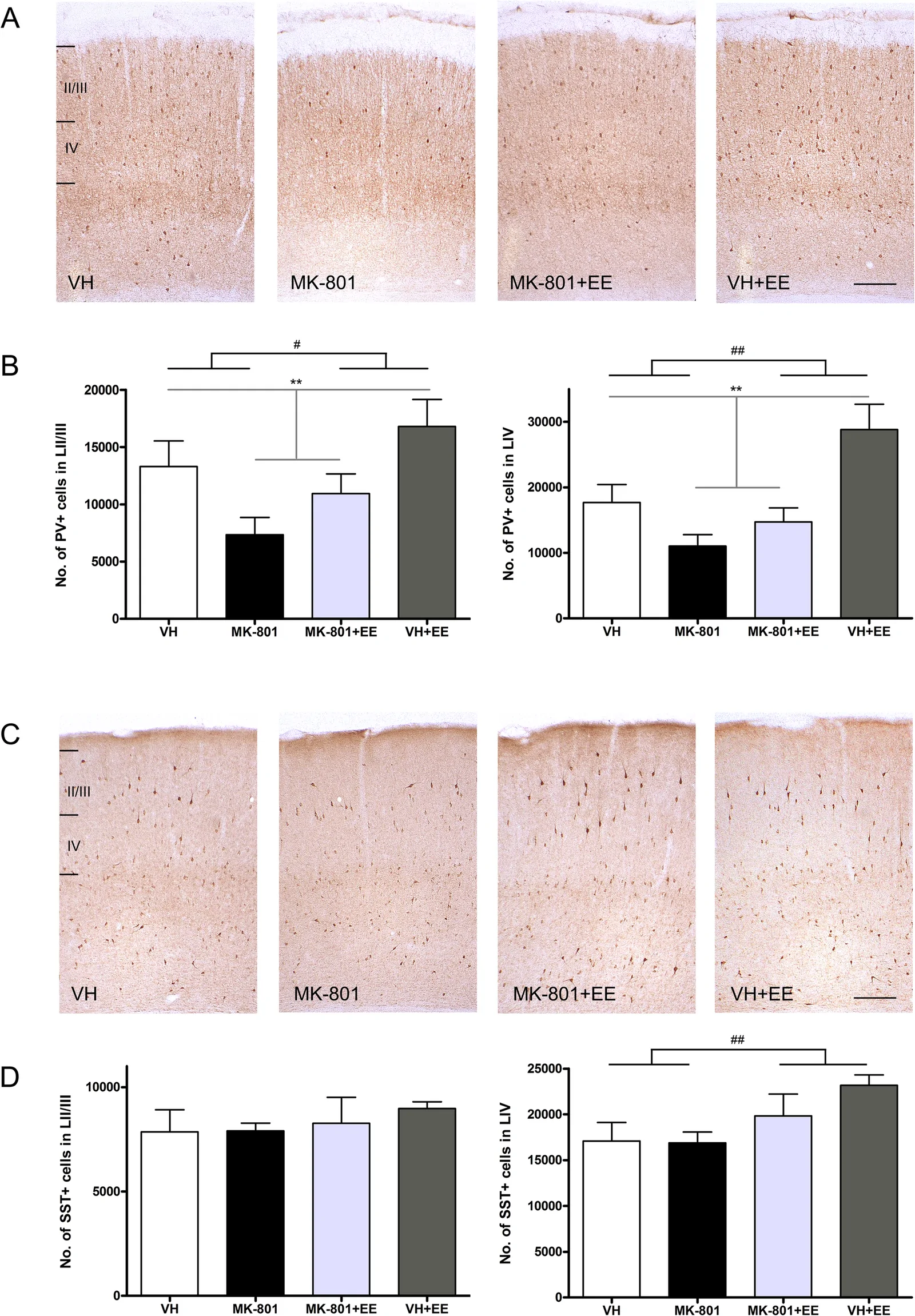
Early NMDA receptor blockade reduces PV-immunoreactive interneurons in V1 and environmental enrichment increases interneuron numbers. Effects of early postnatal MK-801 administration and adult environmental enrichment (EE) on interneuron populations in the primary visual cortex (V1). (**A**) Representative photomicrographs of PV+ interneurons in layers II/III and IV across experimental groups. (**B**) Stereological quantification of PV-immunoreactive interneurons in layers II/III and IV. MK-801 treatment significantly reduced the estimated number of PV-immunoreactive cells in both layers, whereas EE increased PV-immunoreactivity across groups. (**C**, **D**) Quantification of SST-immunoreactive interneurons in layers II/III and IV. SST-immunoreactive cell numbers were not significantly affected by MK-801 treatment, while EE increased the estimated number of SST-immunoreactive cells in layer IV. Data are presented as mean ± SD. Statistical analysis was performed using two-way ANOVA with treatment and housing as factors. No significant treatment × housing interactions were detected for interneuron populations. Treatment effect: * *p* < 0.05, ** *p* < 0.01; Housing effect: ^#^ *p* < 0.05, ^##^ *p* < 0.01. Scale bar = 200 μm

Early postnatal NMDA receptor blockade with MK-801 induced a marked reduction in inhibitory interneuron populations within the V1, with clear subtype-specific effects. A significant main effect of treatment was observed for PV+ interneurons in layers II/III (F(1,19) = 10.2, *p* = 0.005) and IV (F(1,19) = 14.6, *p* = 0.001). Quantitatively, MK-801 reduced the estimated number of PV-immunoreactive cells by approximately 45% in layer II/III and 38% in layer IV (Fig. 2A, B; Table 1), indicating a marked reduction of this interneuron subtype. In contrast, SST+ interneurons were not significantly affected by MK-801 treatment in either layer (Fig. 2C, D).

**Table 1:** Stereological estimates of the number of PV-immunoreactive and SST-immunoreactive interneurons in the primary visual cortex (V1)

	VH	MK-801	MK-801 + EE	VH + EE	Treatment	Housing
*PV+*						
Layer II/III	13302 (5457)	7337 (3699)	10933 (4194)	16809 (5733)	**0.005**	**0.024**
Layer IV	17679 (6793)	11033 (4283)	14705 (5331)	28827 (9500)	**0.001**	**0.006**
*SST+*						
Layer II/III	7852 (2596)	7906 (900)	8269 (3058)	8970 (805)	0.959	0.156
Layer IV	17097 (4980)	16888 (2907)	19828 (5850)	23182 (2814)	0.528	**0.002**
*Volume*						
Layer II/III	7.50 (2.17)	6.83 (0.46)	8.24 (0.46)	7.20 (1.03)	0.747	0.298
Layer IV	6.73 (1.98)	6.06 (0.51)	7.26 (0.36)	6.38 (0.94)	0.826	0.361

Data are represented as mean (SD). Statistical analysis was performed using two-way ANOVA with treatment (saline vs. MK-801) and housing (standard vs. EE) as main factors. Significant main effects are indicated in bold; significant interactions are reported in the Results section. *Abbreviations*: EE, Enriched Environment; PV, parvalbumin, SST, somatostatin, VH, vehicle (saline 0.9%).

Exposure to short-term adult EE exerted subtype-dependent effects. EE significantly increased the number of PV-immunoreactive cells in both layers II/III (F(1,19) = 6.0, *p* = 0.024) and IV (F(1,19) = 9.6, *p* = 0.006). In MK-801-treated animals, EE was associated with increases of approximately 49% and 33% in the estimated number of PV-immunoreactive cells in layers II/III and IV, respectively. EE also increased the estimated number of PV-immunoreactive cells in vehicle-treated animals (26% in layer II/III and 63% in layer IV), indicating that the effects of EE were not restricted to MK-801-treated animals but occurred across groups, thereby reducing the difference between MK-801-treated and control animals. Regarding SST-immunoreactive interneurons, EE had a significant effect in layer IV (F(1,19) = 13.7, *p* = 0.002), with a ~ 17% increase in MK-801-treated animals and ~ 36% in controls, whereas only modest, non-significant changes were observed in layer II/III. No significant treatment × housing interactions were detected for any interneuron population, indicating that EE exerts a general facilitatory effect on interneuron-marker expression rather than a selective reversal of MK-801-induced alterations.

Stereological coefficients of error were generally low and comparable across groups, although they tended to be higher for the estimated number of PV-immunoreactive cells and in MK-801-treated groups, likely reflecting greater biological variability induced by early postnatal treatment. Individual stereological estimates with coefficients of error (CE), coefficients of variation (CV) for each experimental group, and number of sections analyzed per animal are provided in the Supplementary Material.

**Fig. 3:**
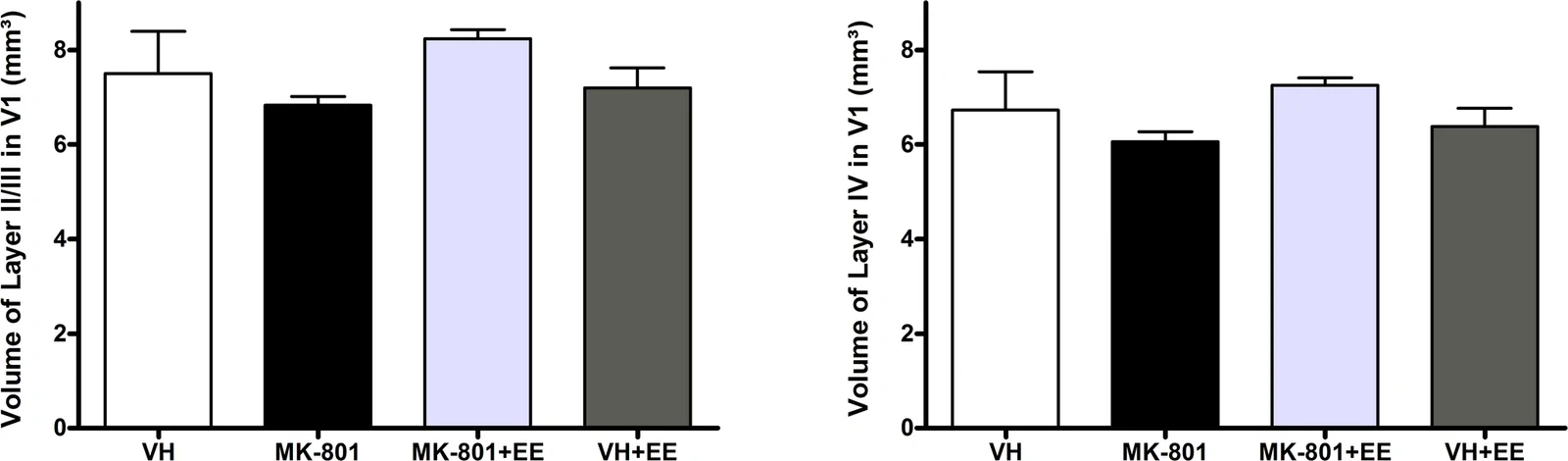
V1 layer volumes are not significantly affected by MK-801 treatment or environmental enrichment. Volume estimates of layers II/III and IV in the primary visual cortex (V1). No significant main effects of treatment or environmental enrichment (EE) were observed in either layer. Descriptive trends indicated slightly lower volumes in MK-801-treated animals and a modest increase with EE, but these differences did not reach statistical significance. Data are presented as mean ± SD. Statistical analysis was performed using two-way ANOVA

Two-way ANOVA revealed no main effects of treatment or housing condition in the volume of either layer II/III (treatment: F(1,19) = 0.107, *p* = 0.747; housing: F(1,19) = 1.147, *p* = 0.298) or layer IV (treatment: F(1,19) = 0.050, *p* = 0.826; housing: F(1,19) = 0.877, *p* = 0.361) of V1. Descriptively, MK-801 animals showed slightly lower volume estimates compared to controls, while EE tended to increase volume in MK-801 animals but not in controls; however, these differences did not reach statistical significance (Fig. 3; Table 1).

Together, these findings indicate that early NMDA receptor blockade preferentially affects PV-immunoreactive interneurons within the broader GABAergic population, while largely sparing SST-immunoreactive interneurons. Short-term adult EE increased interneuron-marker immunoreactivity across groups rather than selectively reversing MK-801-induced alterations.

### Environmental enrichment enhances synaptic plasticity signaling without reversing persistent NMDA receptor deficits

Western blot analysis was conducted to assess molecular correlates of excitatory–inhibitory balance and synaptic plasticity, revealing persistent molecular alterations following MK-801 treatment alongside selective modulation by EE.

**Fig. 4:**
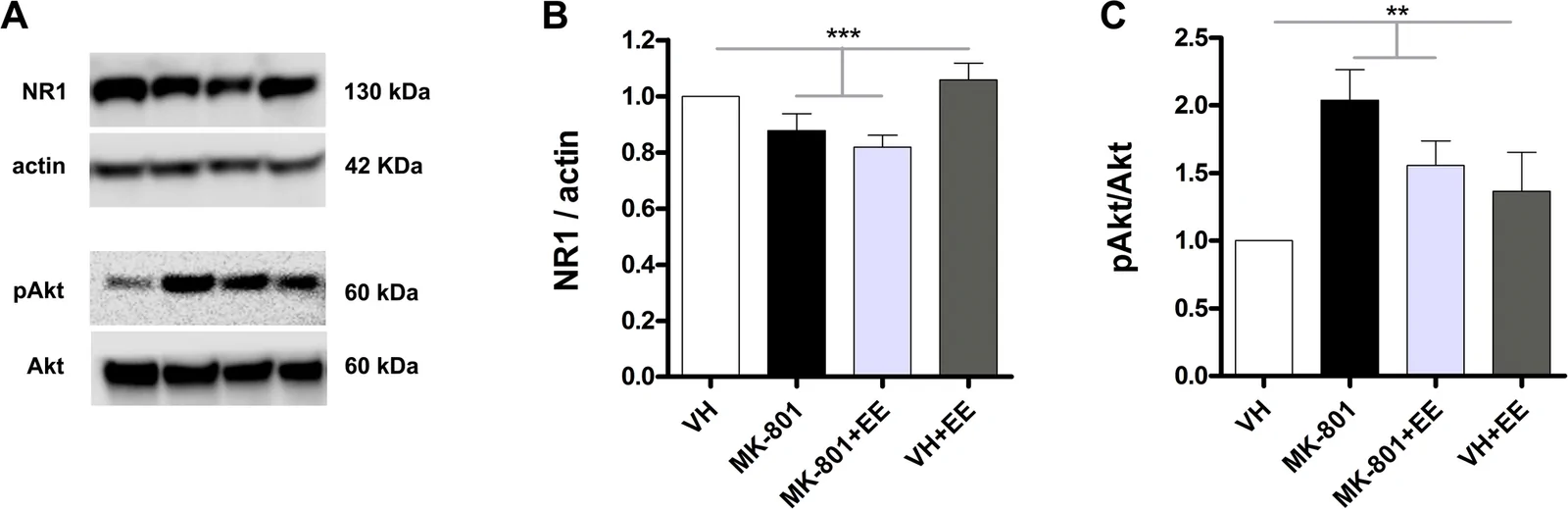
Persistent reduction of NR1 expression and modulation of Akt signaling following environmental enrichment. Western blot analysis of NMDA receptor and intracellular signaling pathways in V1. (**A**) Representative immunoblots for NR1 and pAkt/Akt. (**B**) Quantification of NR1 expression showing a significant reduction following MK-801 treatment, with no effect of environmental enrichment (EE). (**C**) Quantification of pAkt/Akt ratio. MK-801 treatment increased Akt phosphorylation, while EE modulated this effect, as indicated by a significant treatment × housing interaction. Data are presented as mean ± SD. Statistical analysis was performed using two-way ANOVA. Treatment effect: ** *p* < 0.01, *** *p* < 0.001

A significant main effect of MK-801 treatment was observed for the NMDA receptor NR1 subunit (F(1,20) = 14.8, *p* = 0.001), indicating a sustained reduction in receptor expression (Fig. 4). Additionally, MK-801 significantly increased Akt phosphorylation (F(1,20) = 12.6, *p* = 0.002), consistent with dysregulated intracellular signaling (Fig. 4). While EE housing alone had no significant effect on pAkt/Akt ratio (F(1,20) = 0.023, *p* = 0.880), a significant treatment × housing interaction was detected (F(1,20) = 4.5, *p* = 0.048), indicating that EE modulates Akt signaling in a treatment-dependent manner, consistent with a partial normalization of MK-801-induced dysregulation. No significant MK-801 treatment effects were detected for PSD95, pERK/ERK, or GABA_A_ receptor β2/3 subunits.

**Fig. 5:**
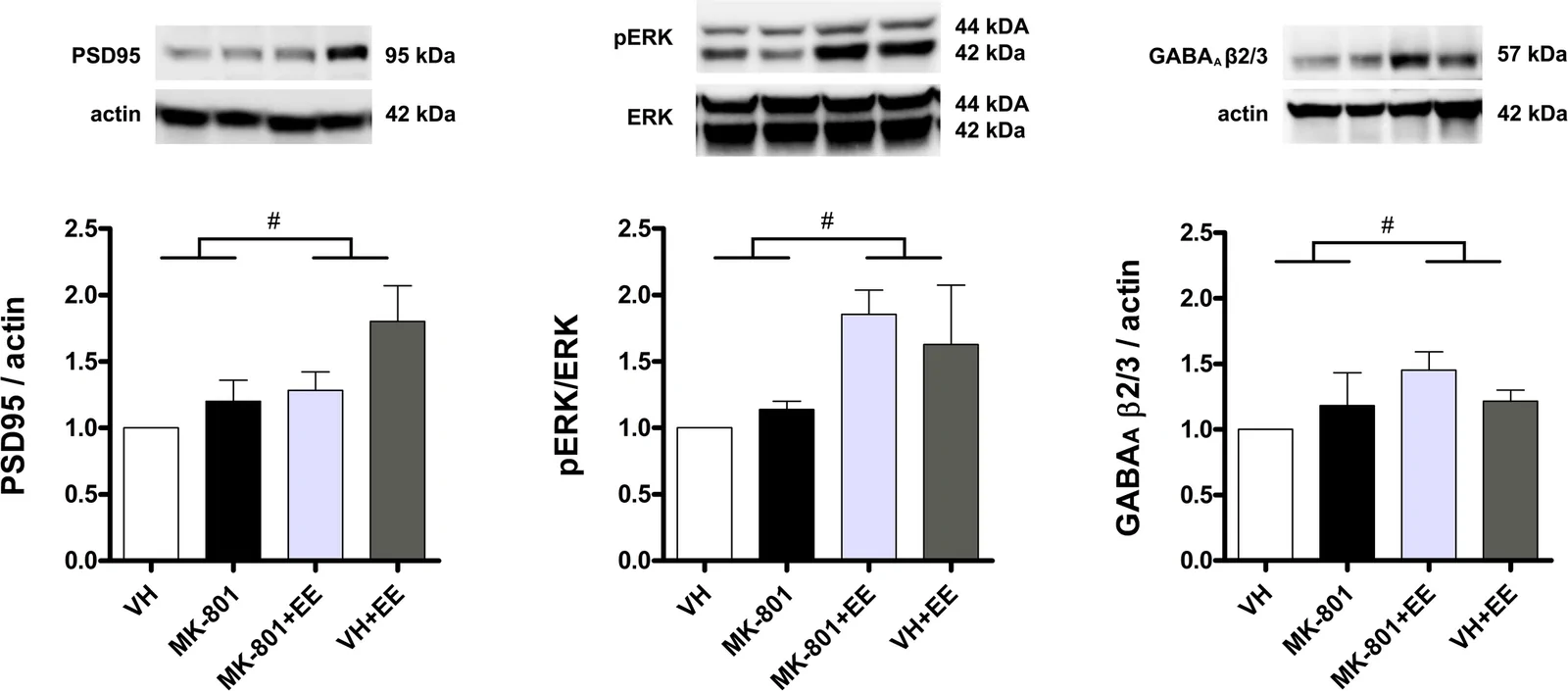
Environmental enrichment modulates synaptic plasticity-related proteins in V1. Effects of environmental enrichment (EE) on synaptic plasticity markers in V1. (**A**) Representative immunoblots for PSD95, pERK/ERK, and GABA_A_ receptor β2/3 subunits. (**B**–**D**) Quantification of protein expression levels. EE significantly increased PSD95 expression, ERK phosphorylation, and GABA_A_ β2/3 subunit levels. A significant treatment × housing interaction was observed for PSD95, indicating a greater effect of EE in saline-injected animals. Data are presented as mean ± SD. Statistical analysis was performed using two-way ANOVA. Housing effect: ^#^ *p* < 0.05

In contrast, after a short-term EE intervention in adulthood, GABA_A_ receptor β2/3 subunits showed increased expression with EE (F(1,20) = 4.808, *p* = 0.040) (Fig. 5). Notably, EE had no effect on NR1 expression (F(1,20) ≈ 0.000, *p* = 0.994), but significantly increased PSD95 (F(1,20) = 6.7, *p* = 0.017) and pERK/ERK (F(1,20) = 7.6, *p* = 0.012) (Fig. 5). A significant treatment × housing interaction was also observed for PSD95 (F(1,20) = 4.4, *p* = 0.049), with a greater effect of EE in saline-injected animals. These findings suggest that EE enhances inhibitory receptor levels and, despite persistent NMDA receptor and PSD95 expression deficits, promotes activation of downstream excitatory signaling pathways.

## Discussion

Early postnatal NMDA receptor blockade with MK-801 reduced the estimated number of PV-immunoreactive cells in layers II/III and IV of the primary visual cortex, whereas SST-immunoreactive cells were largely unaffected. Adult EE increased PV immunoreactivity and, to a lesser extent, SST immunoreactivity in layer IV. However, no significant treatment × housing interaction was detected for interneuron populations, indicating that EE did not selectively reverse MK-801-induced alterations but instead exerted a general facilitatory effect on interneuron-marker expression. At the molecular level, EE enhanced plasticity-related signaling, including PSD95 and ERK phosphorylation, and increased GABA_A_ receptor β2/3 subunit expression. In addition, EE modulated intracellular signaling pathways, partially normalizing MK-801-induced alterations in Akt phosphorylation. However, EE did not alter the reduction in the NMDA receptor NR1 subunit and was not associated with increased PSD95 levels in MK-801-treated animals, indicating that core aspects of NMDA receptor hypofunction persist in V1 despite environmental intervention. Importantly, because interneuron populations were identified by PV and SST immunolabeling, the present findings should be interpreted as changes in the number of immunoreactive cells rather than definitive evidence of neuronal loss. In particular, PV is an activity-dependent calcium-binding protein whose expression is closely linked to interneuron functional state and network activity (Patz 2004). Consequently, the reduction in PV-immunoreactive cells following developmental MK-801 exposure, and their increase following EE, most likely reflect alterations in PV expression and interneuron phenotype rather than loss or generation of cortical interneurons. Together, these findings suggest that EE engages molecular pathways associated with synaptic plasticity and modulates inhibitory circuit function, indicating that experience-dependent plasticity remains possible in the primary visual cortex even after early-life NMDA receptor disruption.

Previous studies have demonstrated that perinatal or early life NMDA receptor blockade with MK-801 produces long-lasting deficits in inhibitory interneurons and associated synaptic pathways, with cell type–specific vulnerability (Abekawa et al. 2007; Coleman et al. 2009; Li et al. 2015; Castillo-Gómez et al. 2017). Mechanistically, transient NMDAR blockade during development likely induces apoptotic vulnerability and network hyperexcitability due to disinhibited glutamatergic output, contributing to schizophrenia-related phenotypes (Xi et al. 2009; Hasan et al. 2011). The pronounced susceptibility of PV+ interneurons may further reflect the high sensitivity of fast-spiking GABAergic neurons to disruptions in NMDA receptor–mediated excitatory drive during critical developmental windows, potentially linked to their distinct NMDA receptor functional properties (Xi et al. 2009; Hasan et al. 2011). The resulting disinhibition of pyramidal neurons may lead to local hyperexcitability, impairing gamma oscillations, sensory gain control, and cortical signal integration—phenotypes commonly observed in schizophrenia models (Volman et al. 2011; Fujihara et al. 2015; Marín 2024). In humans, PV+ dysfunction has been linked to impaired gamma oscillations and deficits in visuospatial working memory, supporting a shared circuit-level substrate across sensory and associative networks (Fish et al. 2021; Dienel et al. 2025). However, most studies in animal models have focused on the prefrontal cortex and hippocampus, whereas our work extends these findings by directly examining interneuron populations in the primary visual cortex in the neurodevelopmental MK-801 model, a region that has not been characterized. The selective vulnerability of PV+ interneurons to early-life MK801 administration, in contrast to SST+ cells in the visual cortex, underscores subtype-specific mechanisms of plasticity and resilience. SST+ interneurons, which predominantly mediate dendritic inhibition and fine-tune receptive field properties, may be less dependent on early NMDA receptor–mediated trophic signaling in sensory circuits, relying instead on activity-dependent mechanisms involving kainate receptors and metabotropic glutamate receptors (Stachniak et al. 2023). This contrasts with associative cortices, where SST+ deficits have been observed in this model (Murueta-Goyena et al. 2020), consistent with findings in humans showing reduced interneuron-related mRNA expression associated with impaired low‑frequency oscillations, cortico-cortical feedback, and cognitive dysfunction (Van Derveer et al. 2021; Dienel et al. 2025). However, the impact on these interneurons on sensory cortices remains less well understood.

Previous work from our group using the same MK-801 neurodevelopmental model demonstrated that short-term environmental enrichment in adulthood ameliorated cognitive deficits induced by early NMDA receptor blockade. Specifically, EE restored spatial learning and memory performance in the Morris Water Maze and improved object recognition and object–place associative memory impairments, while also attenuating locomotor and anxiety-like behaviors in the Open Field test (Murueta-Goyena et al. 2018, 2019). These behavioral improvements were paralleled by enhanced number of GABAergic interneuron markers in hippocampus and prefrontal cortex, and by restoration of hippocampal–prefrontal synaptic plasticity and NMDA receptor subunit expression (Murueta-Goyena et al. 2019). Consistently, EE has been shown to normalize interneuron-related alterations in limbic and associative circuits, including restoration of somatostatin-expressing interneurons after short-term enrichment, (Murueta-Goyena et al. 2020), and partial recovery of GABAergic immunoreactivity (Murueta-Goyena et al. 2018). These alterations align with evidence from the human cortex, where reduced PV expression is thought to reflect impaired inhibitory function rather than interneuron loss (Fish et al. 2021; Marín 2024). In this work, the increase in PV-immunoreactive cells observed in V1 supports a role for EE in promoting experience-dependent plasticity of inhibitory circuits. Our findings extend previous work in hippocampus and prefrontal cortex (Ji et al. 2017; Murueta-Goyena et al. 2018; Donato et al. 2025), as well as studies demonstrating enhanced visual cortical plasticity in adult rodents after EE (Mainardi et al. 2010; Tognini et al. 2012; Baroncelli et al. 2016), indicating that PV+ interneurons are particularly sensitive to EE, supporting a region-specific modulation of inhibitory circuit integrity.

Molecular findings further suggest that compensatory mechanisms may emerge to counteract early NMDA receptor dysfunction, as EE not only increases the number of PV-immunoreactive cells but also engages molecular substrates of synaptic and intracellular plasticity, including enhanced ERK phosphorylation, and upregulated GABA_A_ β2/3 subunits, despite persistent reductions in NR1 expression. These changes provide a potential mechanistic link between previously observed behavioral recovery and region-specific circuit remodeling (Möller et al. 2024). In this context, the modulation of inhibitory circuit integrity and promotion of synaptic plasticity in sensory cortex may contribute to improved integration of sensory and cognitive information, consistent with our previous findings showing that EE enhances performance in spatial navigation and memory tasks in this model that depend on visual inputs (Murueta-Goyena et al. 2018, 2019). However, because visual function was not directly assessed, the contribution of V1 plasticity to the behavioral improvements previously reported in this model remains speculative. Moreover, these behavioral effects parallel previously reported molecular adaptations, including enhanced neurotrophic signaling and modification of NMDA receptor subunit expression in hippocampus and prefrontal cortex (Murueta-Goyena et al. 2019, 2020), suggesting that enriched experience engages both associative and sensory networks to compensate for early NMDA receptor hypofunction.

Reduced NR1 expression persists in the visual cortex of previously MK-801-treated animals and is not modulated by enrichment, while PSD95 fails to show the EE-induced increase observed in vehicle animals. In contrast, PV+ interneuron numbers increase after EE despite the persistence of these postsynaptic and NMDA-related deficits, indicating a dissociation between inhibitory circuit remodeling and full restoration of glutamatergic synaptic markers. This pattern contrasts with that observed in hippocampus and medial prefrontal cortex, where enrichment can restore NR1 levels (Sun et al. 2010; Murueta-Goyena et al. 2019), highlighting region- and timing-dependent limits of plasticity. Nevertheless, EE engages intracellular signaling pathways associated with synaptic plasticity, including ERK activation, which is involved in dendritic spine maturation, synaptic stabilization, and neuronal survival (Lee et al. 2009; Yoshii et al. 2011). In parallel, EE increases GABA_A_ β2/3 subunit expression, indicating enhanced inhibitory postsynaptic receptor function and contributing to the restoration of excitation–inhibition balance (Beston et al. 2010). Consistent with this, Akt hyperphosphorylation—previously reported in other brain regions in this model (Takagi et al. 2015; Murueta-Goyena et al. 2020) and linked to GABA_A_ receptor trafficking via increased receptor insertion at the plasma membrane (Wang et al. 2003)—is reduced by EE, suggesting attenuation of MK-801-associated dysregulation and stabilization of inhibitory synaptic transmission. Together, these findings support the idea that EE promotes inhibitory network remodeling and synaptic plasticity in V1 through intracellular signaling and receptor trafficking mechanisms, even in the absence of full recovery of NMDA receptor expression.

Several limitations should be considered when interpreting the present findings. First, the study did not include direct functional assessment of the primary visual cortex. Consequently, the functional significance of the observed cellular and molecular alterations remains to be established, and the contribution of V1 plasticity to the behavioral improvements previously reported in this model should be interpreted with caution. Second, stereological analyses were restricted to layers II/III and IV, because of their major role in thalamocortical input processing and intracortical visual integration. However, layers V/VI contain corticofugal and feedback projection circuits that may also be vulnerable to early NMDA receptor hypofunction. Therefore, the present findings cannot be generalized to the full laminar organization of V1, and future studies should examine whether infragranular layers show comparable or distinct interneuron and molecular alterations. Third, the study focused specifically on the interneuron subtypes most consistently implicated in schizophrenia and did not assess pyramidal neurons. Although the absence of significant V1 layer-volume changes, together with previous reports showing preserved total neuronal number following MK-801 exposure (Liu et al. 2008; Murueta-Goyena et al. 2018), suggests that excitatory neuronal populations may be relatively spared, subtle changes in pyramidal neuron density, dendritic architecture, or excitatory synaptic organization cannot be excluded. Fourth, unlike stereological estimates, Western blot analyses were performed on whole dissected V1 tissue and therefore lacked laminar and cell-type resolution. As a result, the molecular changes observed cannot be attributed to specific cortical layers or neuronal populations. Finally, only male rats were included in the study. This limits the generalizability of the findings, particularly given known sex differences in interneuron maturation, NMDA receptor signaling, responses to environmental enrichment, and schizophrenia epidemiology. Future studies should include both sexes to determine whether the present V1 alterations and EE-related effects are sex dependent.

## Conclusions

Our findings support a multi-level mechanistic framework in which early NMDA receptor hypofunction disrupts inhibitory network organization and intracellular signaling, leading to a selective reduction of PV-immoreactive interneurons in the primary visual cortex. Environmental enrichment in adulthood engages molecular pathways associated with synaptic plasticity and increases PV-immunoreactive cell estimates. Notably, these effects occur despite persistent reductions in NR1 and in the absence of PSD95 increase, suggesting that enrichment-related changes may occur without full restoration of NR1 expression or excitatory postsynaptic organization. This convergence of cellular, synaptic, and signaling-level changes may contribute to the mechanisms underlying previously reported behavioral improvements. Overall, these results indicate that early-life MK-801 exposure induces long-lasting alterations in visual cortical circuitry, while experience-dependent plasticity in adulthood remains capable of modulating these alterations. These findings highlight potential translational avenues for interventions targeting inhibitory function and synaptic plasticity in schizophrenia-related neurodevelopmental disorders but warrant further functional and mechanistic validation. 

## Supplementary Information

Below is the link to the electronic supplementary material.

Supplementary Material 1

## Data Availability

The data generated from stereological estimates is provided in the Supplementary Material. Additional data underlying the figures are available upon reasonable request.
