# Supplementary material for: Environmental enrichment modulates parvalbumin interneuron deficits and plasticity-related signaling after early postnatal NMDA receptor hypofunction in male rat visual cortex
Source: Brain Struct Funct. 2026 Aug 4;231(7):114. doi: 10.1007/s00429-026-03168-8 (PMC13437568; doi:10.1007/s00429-026-03168-8)
Supplement: Supplementary file 1 [file 429_2026_3168_MOESM1_ESM.docx]

**Environmental enrichment modulates parvalbumin interneuron deficits and plasticity-related signaling after early postnatal NMDA receptor hypofunction in male rat visual cortex**

**Authors:**

Ane Murueta-Goyena, PhD^1,2^; Naiara Ortuzar, PhD^1,2^; Susana Bulnes, PhD^1,2^; José Vicente Lafuente, MD, PhD^1,2^; Harkaitz Bengoetxea, PhD^1,2^

**Affiliations**:

^1^Department of Neurosciences, Faculty of Medicine and Nursery, University of the Basque Country (EHU), Leioa, 48940, Bizkaia

^2^Neurodegenerative diseases group, Biobizkaia Health Research Institute, Barakaldo, 48903, Bizkaia

**Corresponding author:**

Ane Murueta-Goyena, PhD

Barrio Sarriena s/n, Leioa (Bizkaia)

Tlfn: +34 94 601 27 55

ane.muruetagoyena@ehu.eus

**CONTENT:**

**Supplementary Table S1.** Individual stereological estimates, coefficients of error (CE), and number of analyzed sections per animal for parvalbumin-positive (PV+) interneurons

**Supplementary Table S2.** Individual stereological estimates, coefficients of error (CE), and number of analyzed sections per animal for somatostatin-positive (SST+) interneurons

**Supplementary Table S3.** Coefficients of variation (CV) of stereological cell number estimates of parvalbumin- and somatostatin-positive interneurons across experimental groups

**Supplementary Table S1.** Individual stereological estimates, coefficients of error (CE), and number of analyzed sections per animal for parvalbumin-positive (PV+) interneurons

|  |  |  |  | Layer II/III | | |  | | Layer IV | | |
| --- | --- | --- | --- | --- | --- | --- | --- | --- | --- | --- | --- |
| Treatment | Housing | Animal ID | No. of sections | N | CE |  | | N | | CE |  |
| VH | SC | 1 | 5 | 12303 | 0.10 |  | | 15723 | | 0.12 |  |
| VH | SC | 2 | 5 | 12303 | 0.09 |  | | 16580 | | 0.09 |  |
| VH | SC | 3 | 5 | 22508 | 0.08 |  | | 30606 | | 0.06 |  |
| VH | SC | 4 | 6 | 16251 | 0.08 |  | | 17040 | | 0.07 |  |
| VH | SC | 5 | 4 | 7241 | 0.20 |  | | 10268 | | 0.17 |  |
| VH | SC | 6 | 5 | 9211 | 0.11 |  | | 15857 | | 0.13 |  |
| MK-801 | SC | 7 | 5 | 6910 | 0.13 |  | | 9675 | | 0.15 |  |
| MK-801 | SC | 8 | 4 | 3621 | 0.18 |  | | 10403 | | 0.16 |  |
| MK-801 | SC | 9 | 5 | 5790 | 0.18 |  | | 5790 | | 0.09 |  |
| MK-801 | SC | 10 | 5 | 6450 | 0.15 |  | | 10399 | | 0.15 |  |
| MK-801 | SC | 11 | 6 | 14474 | 0.16 |  | | 18881 | | 0.15 |  |
| MK-801 | SC | 12 | 5 | 6778 | 0.20 |  | | 11054 | | 0.14 |  |
| MK-801 | EE | 13 | 6 | 11119 | 0.05 |  | | 11907 | | 0.06 |  |
| MK-801 | EE | 14 | 6 | 9342 | 0.09 |  | | 12830 | | 0.13 |  |
| MK-801 | EE | 15 | 4 | 9216 | 0.08 |  | | 15536 | | 0.08 |  |
| MK-801 | EE | 16 | 5 | 10855 | 0.14 |  | | 14671 | | 0.13 |  |
| MK-801 | EE | 17 * | 5 | 6316 | 0.36 |  | | 8816 | | 0.36 |  |
| MK-801 | EE | 18 | 6 | 18751 | 0.09 |  | | 24474 | | 0.09 |  |
| VH | EE | 19 | 5 | 13619 | 0.10 |  | | 17763 | | 0.09 |  |
| VH | EE | 20 | 5 | 10527 | 0.18 |  | | 19343 | | 0.17 |  |
| VH | EE | 21 | 7 | 23487 | 0.06 |  | | 37697 | | 0.07 |  |
| VH | EE | 22 | 6 | 13158 | 0.16 |  | | 25395 | | 0.15 |  |
| VH | EE | 23 | 6 | 24276 | 0.09 |  | | 40527 | | 0.08 |  |
| VH | EE | 24 | 6 | 15789 | 0.09 |  | | 32237 | | 0.08 |  |

*Data point excluded from statistical analysis due to coefficient of error (CE) exceeding the acceptable threshold (CE = 0.36) for stereological estimates.

*Abbreviations:* CE, coefficient of error; EE, enriched environment; N, stereological estimates of interneuron number per animal; SC, Standard Conditions; VH, vehicle (0.9% NaCl).

**Supplementary Table S2.** Individual stereological estimates, coefficients of error (CE), and number of analyzed sections per animal for somatostatin-positive (SST+) interneurons

|  |  |  |  | Layer II/III | |  | Layer IV | |
| --- | --- | --- | --- | --- | --- | --- | --- | --- |
| Treatment | Housing | Animal ID | No. of sections | N | CE |  | N | CE |
| VH | SC | 1 | 5 | 12303 | 0.08 |  | 15723 | 0.07 |
| VH | SC | 2 | 5 | 12303 | 0.10 |  | 16580 | 0.10 |
| VH | SC | 3 | 5 | 22508 | 0.06 |  | 30606 | 0.06 |
| VH | SC | 4 | 6 | 16251 | 0.09 |  | 17040 | 0.09 |
| VH | SC | 5 | 4 | 7241 | 0.10 |  | 10268 | 0.09 |
| VH | SC | 6 | 5 | 9211 | 0.06 |  | 15857 | 0.13 |
| MK-801 | SC | 7 | 5 | 6910 | 0.08 |  | 9675 | 0.06 |
| MK-801 | SC | 8 | 4 | 3621 | 0.10 |  | 10403 | 0.09 |
| MK-801 | SC | 9 | 5 | 5790 | 0.13 |  | 5790 | 0.17 |
| MK-801 | SC | 10 | 5 | 6450 | 0.06 |  | 10399 | 0.07 |
| MK-801 | SC | 11 | 6 | 14474 | 0.07 |  | 18881 | 0.05 |
| MK-801 | SC | 12 | 5 | 6778 | 0.06 |  | 11054 | 0.07 |
| MK-801 | EE | 13 | 6 | 11119 | 0.06 |  | 11907 | 0.06 |
| MK-801 | EE | 14 | 6 | 9342 | 0.06 |  | 12830 | 0.08 |
| MK-801 | EE | 15 | 4 | 9216 | 0.09 |  | 15536 | 0.09 |
| MK-801 | EE | 16 | 5 | 10855 | 0.06 |  | 14671 | 0.07 |
| MK-801 | EE | 17 | 5 | 6316 | 0.10 |  | 8816 | 0.15 |
| MK-801 | EE | 18 | 6 | 18751 | 0.09 |  | 24474 | 0.05 |
| VH | EE | 19 | 5 | 13619 | 0.09 |  | 17763 | 0.07 |
| VH | EE | 20 | 5 | 10527 | 0.08 |  | 19343 | 0.07 |
| VH | EE | 21 | 7 | 23487 | 0.08 |  | 37697 | 0.07 |
| VH | EE | 22 | 6 | 13158 | 0.05 |  | 25395 | 0.07 |
| VH | EE | 23 | 6 | 24276 | 0.06 |  | 40527 | 0.05 |
| VH | EE | 24 | 6 | 15789 | 0.10 |  | 32237 | 0.08 |

*Abbreviations:* CE, coefficient of error; EE, enriched environment; N, stereological estimates of interneuron number per animal; SC, Standard Conditions; VH, vehicle (0.9% NaCl).

**Supplementary Table S3.** Coefficients of variation (CV) of stereological cell number estimates of parvalbumin- and somatostatin-positive interneurons across experimental groups.

|  | **Layer** | **VH (%)** | **MK-801 (%)** | **MK-801+EE (%)** | **VH+EE (%)** | **p-value** |  |  |
| --- | --- | --- | --- | --- | --- | --- | --- | --- |
| PV+ | II/III | 41.0 | 50.4 | 33.3 | 34.1 | 0.627 |  |  |
|  | IV | 38.4 | 38.8 | 31.6 | 33.0 | 0.141 |  |  |
| SST+ | II/III | 33.1 | 11.4 | 37.0 | 9.0 | 0.103 |  |  |
|  | IV | 29.1 | 17.2 | 29.5 | 12.1 | 0.610 |  |  |

Data represent the coefficient of variation (CV = SD/mean × 100) of stereological cell number estimates within each experimental group. p-values were derived from Brown–Forsythe tests performed on individual animal estimates.
